# Structure and mechanism of a methyltransferase ribozyme

**DOI:** 10.1038/s41589-022-00982-z

**Published:** 2022-03-17

**Authors:** Jie Deng, Timothy J. Wilson, Jia Wang, Xuemei Peng, Mengxiao Li, Xiaowei Lin, Wenjian Liao, David M. J. Lilley, Lin Huang

**Affiliations:** 1 Guangdong Provincial Key Laboratory of Malignant Tumor Epigenetics and Gene Regulation, Guangdong-Hong Kong Joint Laboratory for RNA Medicine, Sun Yat-Sen Memorial Hospital, Sun Yat-Sen University, Guangzhou, China; 2 Medical Research Center, Sun Yat-Sen Memorial Hospital, Sun Yat-Sen University, Guangzhou, China; 3 Cancer Research UK Nucleic Acid Structure Research Group, MSI/WTB Complex, The University of Dundee, Dundee, UK; 4 College of Life Sciences, Guangzhou University, Guangzhou, China; 5 Department of Urology, Sun Yat-Sen Memorial Hospital, Sun Yat-Sen University, Guangzhou, China

**Keywords:** Biocatalysis, RNA

## Abstract

Known ribozymes in contemporary biology perform a limited range of chemical catalysis, but in vitro selection has generated species that catalyze a broader range of chemistry; yet, there have been few structural and mechanistic studies of selected ribozymes. A ribozyme has recently been selected that can catalyze a site-specific methyl transfer reaction. We have solved the crystal structure of this ribozyme at a resolution of 2.3 Å, showing how the RNA folds to generate a very specific binding site for the methyl donor substrate. The structure immediately suggests a catalytic mechanism involving a combination of proximity and orientation and nucleobase-mediated general acid catalysis. The mechanism is supported by the pH dependence of the rate of catalysis. A selected methyltransferase ribozyme can thus use a relatively sophisticated catalytic mechanism, broadening the range of known RNA-catalyzed chemistry.

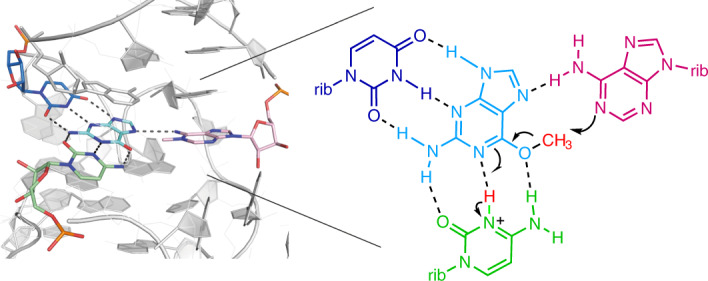

## Abstract (web summary)

The authors present the crystal structure of the MTR1 ribozyme that transfers the methyl group from *O*^6^-methylguanine to an adenine N1 in the target RNA and propose a catalytic mechanism based upon proximity, orientation and general acid catalysis.

## Main

The metabolism of a putative RNA world^1^ would have required RNA molecules that catalyze a broad range of chemical reactions. RNA catalysts (ribozymes)^2^ exist in contemporary biology but catalyze a relatively narrow range of reactions that are limited to phosphoryl transfer reactions and peptide bond formation. Clearly an RNA world would have required ribozymes that facilitate more challenging reactions, such as C–C and C–N bond formation. Yet, the extent of RNA catalysis will be limited by the relatively narrow range of chemical space of RNA compared to proteins restricted to four chemically similar heterocyclic nucleobases, ribose together with its 2-hydroxyl group and a charged phosphodiester linkage with attendant hydrated cations. However, in principle, the chemical functionality of RNA could be greatly expanded by binding small-molecule co-reactants and coenzymes^3–5^. RNA exhibits highly selective binding of ligands; this is exemplified by riboswitches^6,7^, a number of which bind powerful coenzymes, and we have discussed a possible evolutionary relationship between riboswitches and ribozymes^5^.

The potential for RNA catalysis of a much broader range of chemistry has been demonstrated by in vitro evolution of catalytic RNA. By these means, ribozymes have been selected that catalyze a range of bond-forming reactions, including Diels–Alder^8,9^ and aldol condensations^10^, alkylation reactions^11^ and Michael addition^12^. These species offer proof of principle that RNA can expand its range of chemistry, but there has been very little mechanistic investigation of the way in which this is achieved, and the structures of relatively few of these ribozymes have been determined^13,14^. The natural ribozymes that catalyze phosphoryl transfer reactions in general do so by using either metal ion catalysis (for example, the group I intron ribozymes^15–17^) or general acid–base catalysis (for example, the nucleolytic ribozymes, such as the hairpin ribozyme^18–20^), variously using nucleobases, 2′-hydroxyl groups and metal ion-bound water molecules^21^. At the present time, there is very little evidence that selected ribozymes performing a wider range of catalysis use any of these strategies.

**Fig. 1:**
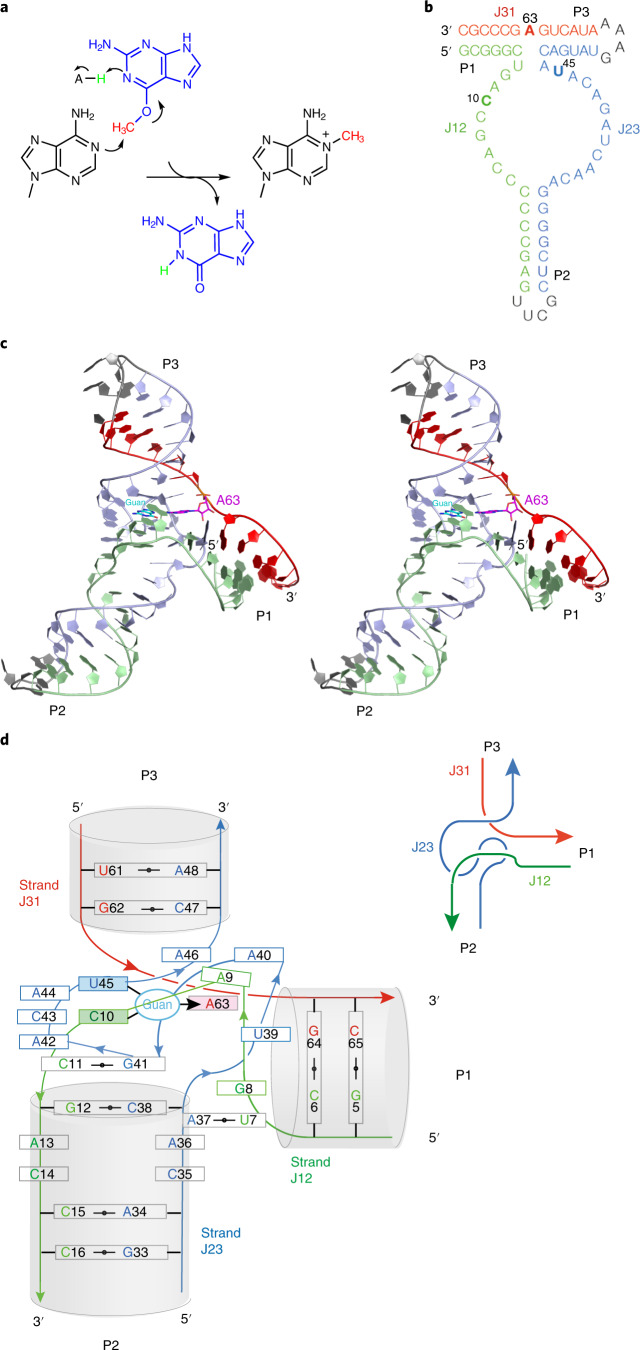
The methyltransferase reaction and the overall structure of the MTR1 ribozyme. **a**, The chemical reaction in which the methyl group of exogenous *O*^6^-methylguanine becomes transferred to N1 of a specific adenine in the RNA. **b**, The sequence of the MTR1 ribozyme as crystallized in this work. The same color scheme is used throughout the paper. The RNA is a three-way junction composed of the three arms P1, P2 and P3. For the crystallographic study, a GNRA tetraloop has been added to the end of the P3 helix so that the entire ribozyme comprises a single RNA strand. Subsections of the strands are named J12 (colored green), J23 (colored blue) and J31 (colored red). **c**, Parallel-eye stereoscopic image of the complete MTR1 ribozyme structure. The bound guanine (guan) is colored cyan, and the methylation target A63 is colored magenta. Note that as the methyl transfer reaction is complete in the crystal, the exogenous base is guanine, and *N*^1^-methyladenine is present at position 63. **d**, Schematic of the structure of the junction with the bound guanine. The inset (top right) is a schematic showing the path of the strands at the junction observed from the minor groove side of the junction.

Methylation of RNA occurs widely^22–24^ and is thought to be an ancient process. Nucleobases and 2-hydroxyl groups are methylated, generally accepting a methyl group from *S*-adenosyl methionine (SAM), catalyzed by a broad range of methyltransferase enzymes. There is a wide range of SAM-binding riboswitches, possibly indicating an ancient connection between RNA and methylation^25^. It has been noted that the manner of ligand binding in the SAM-I riboswitch suggests that it could be converted into a methyltransferase ribozyme relatively easily^4,25^, and thus the riboswitch may have evolved from an ancient ribozyme. Recently, Flemmich et al.^26^ have shown that the prequeuosine_1_ (preQ_1_) riboswitch can exhibit some methyltransferase activity, whereby the methyl group from *O*^6^-methyl preQ_1_ becomes transferred to cytosine N3 with an observed rate on the order of 0.001 min^−1^. Murchie and colleagues^27^ have also very recently selected an RNA that transfers the methyl group of SAM to N7 of a specific guanine of the RNA and determined the crystal structure. Höbartner and colleagues^28^ used in vitro RNA selection to generate a new RNA species called MTR1 that can catalyze the transfer of the methyl group from *O*^6^-methylguanine to N1 of a specific adenine in the RNA (Fig. 1a).

These studies demonstrate that RNA can perform methyl and alkyl transfer reactions, but nothing is known about how this is achieved. We would anticipate that a relatively primitive ribozyme might use proximity and orientation of the reactants to facilitate the reactants for an in-line SN2 reaction, but could such RNAs use other catalytic strategies? To pursue this, we have determined a crystal structure of the MTR1 ribozyme. The RNA was cocrystallized with *O*^6^-methylguanine, but the methyl transfer reaction had gone to completion, so the structure is a product complex that contains bound exogenous guanine, and the target adenine was converted to *N*^1^-methyladenine. Importantly, the structure reveals that the guanine is held in a very precise manner juxtaposed with the target adenine and suggests a mechanism for the methyl transfer reaction that is consistent with experimental data in solution.

## Results

### Crystallization of MTR1

We screened a large number of in vitro-transcribed RNA constructs for crystallization in the presence of *O*^6^-methylguanine and obtained crystals for a one-piece RNA of 69 nucleotides (nt), with hairpin loops at the ends of two helices (Fig. 1b). In this construct, the core structure of the selected ribozyme remains unchanged^28^. The 5′-end of the ribozyme sequence was altered to GCG to improve accuracy of transcription, along with complementary changes to the 3′-end of the RNA. In addition, we included a GAAA loop at the end of the P3 helix to promote crystallization. Diffraction data were collected to a resolution of 2.3 Å, and the structure was solved using the anomalous diffraction of bound barium ions. The coordinates have been deposited at Protein Data Bank (PDB) under ID number 7V9E, and crystallographic statistics are tabulated in Supplementary Table 1.

### The global structure of MTR1

**Extended Data Fig. 1:**
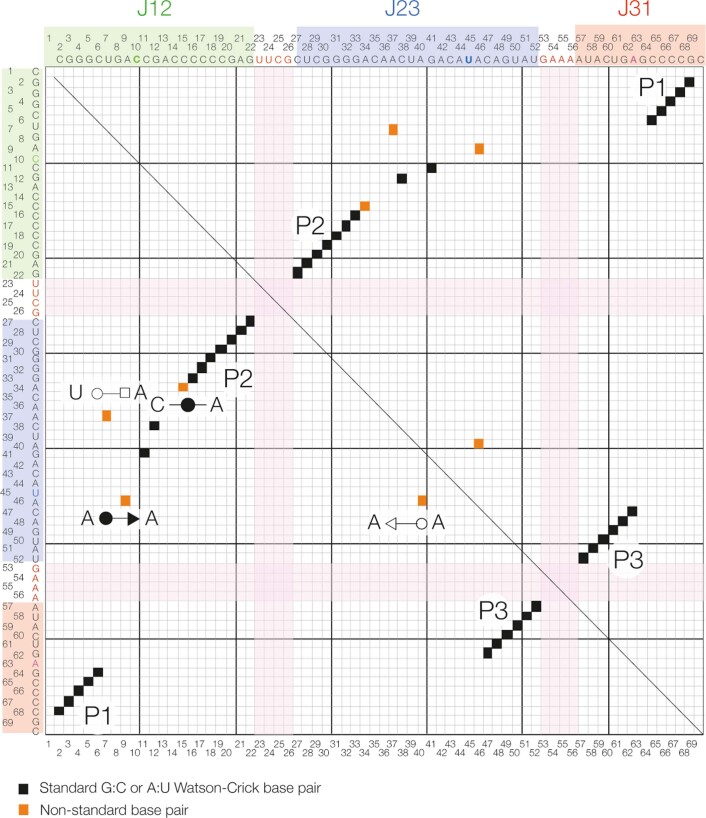
Grid of nucleotide interactions in the MTR1 structure. Grid of nucleotide interactions in the MTR1 structure. Standard G:C or A:U *cis* Watson-Crick base pairs are indicated by black squares. Non-standard base pairs are indicated by orange squares, and the base pairing shown using the Leontis-Westhof designation.

**Extended Data Fig. 2:**
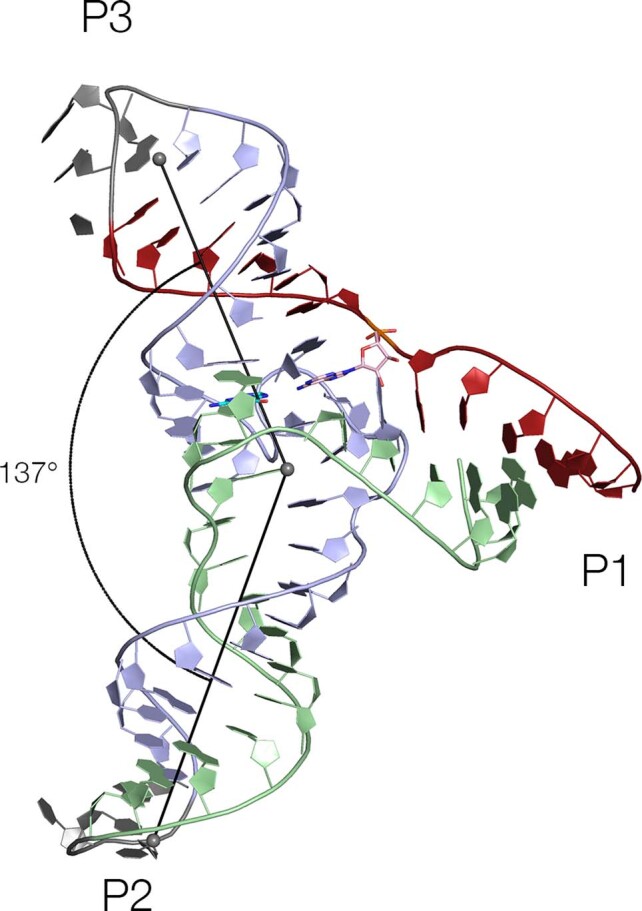
The global structure of the three-way junction of MTR1. The global structure of the three-way junction of MTR1. P2 and P3 are coaxial with the core of the structure, with P1 perpendicular to them. But there is a gentle curvature of the P2-core-P3 axis.

MTR1 has the global structure of a three-way helical junction (Fig. 1c,d). The three arms are designated P1, P2 and P3, and their component strand sections are J12 (green), J23 (blue) and J31 (red). These colors are used throughout. J31 contains the adenine (A63) that is the target of methylation. Using the International Union of Biochemistry nomenclature for junctions^29^, this is a HS_1_HS_5_HS_8_ junction. Reactant binding and the active site of the ribozyme are contained within the core of the junction. The secondary structure of MTR1 is shown in graphical form in Extended Data Fig. 1. P2, the core and P3 are coaxial with continuous base stacking all the way through. However, there is some gentle curvature in the core so that P2 and P3 are mutually bent ~40° from colinearity (Extended Data Fig. 2). The P1 helix is approximately perpendicular to P2 and P3 and not stacked with either.

### The helical arms of the MTR1 junction

**Extended Data Fig. 3:**
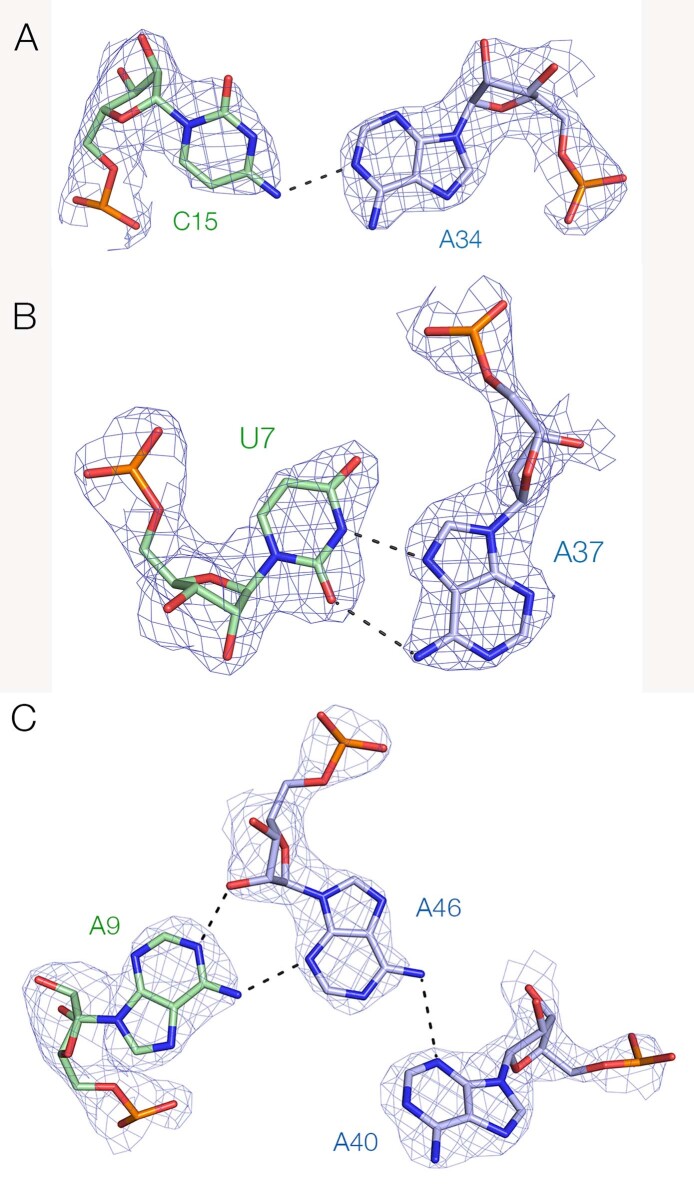
Some non-Watson-Crick nucleobase interactions in the MTR1 structure. Some non-Watson-Crick nucleobase interactions in the MTR1 structure. **a**. The C15:A34 interaction in P2. This is a *cis*-Watson-Crick base pair. **b**. The U7:A37 interaction stacking on the junction-proximal end of P1. This is a *trans*-Watson-Crick-Hoogsteen base pair. **c**. The triple base interaction between A9:A46:A40. This is the fourth plane of interactions in the core of the ribozyme, and sits immediately over the 6-methyl guanine binding site. The 2**F**o**F**c electron density maps are shown contoured at 1.5 σ.

**Extended Data Fig. 4:**
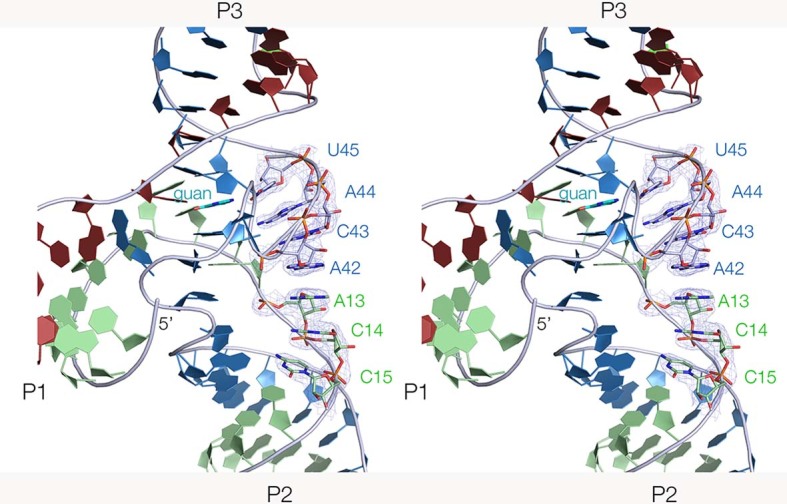
Parallel-eye stereoscopic view of the continuous stack of nucleobases on the side of the core opposite the P1 helix. Parallel-eye stereoscopic view of the continuous stack of nucleobases on the side of the core opposite the P1 helix. This comprises C15, C14, A13 (J12 strand), A42, C43, A44, U45 (J23 strand). The 2**F**o**F**c electron density map is show contoured at 1.5 σ.

The P1 and P3 helices are fully Watson–Crick base-paired helices up to the junction, as expected from the original design used in the selection. By contrast, P2 has several non-Watson–Crick interactions adjacent to the junction, arising from the original design used in the selection. C16•G33 is a standard *cis*-Watson–Crick base pair, but progressing toward the junction, it is followed by C15•A34, a *cis*-Watson–Crick base pair connected by a single hydrogen bond from C15 N4 to A34 N1 (Extended Data Fig. 3a). Thereafter the nucleobases of C14–C35 and A13–A36 are both separated by ~8 Å; that is, this section is essentially a totally unpaired ‘bubble’. However, the nucleobases are all stacked, and A13 and C14 are part of a continuous stack of nucleobases along one side of the junction core (Extended Data Fig. 4). The nucleobases of A42 through to U45 on the J23 strand form a stack, and A42 stacks on A13 of the J12 strand.

The nucleobase of A37 (from J23) is directed out of the helix and forms a *trans*-Watson–Crick–Hoogsteen base pair with U7 (in J12) (Extended Data Fig. 3b). This base pair is stacked on P1 helix, as is nucleotide G8. Finally, the P2 helix is terminated by a *cis*-Watson–Crick G12•C38 base pair before the strands diverge at the junction

### The structure of the core of MTR1

**Fig. 2:**
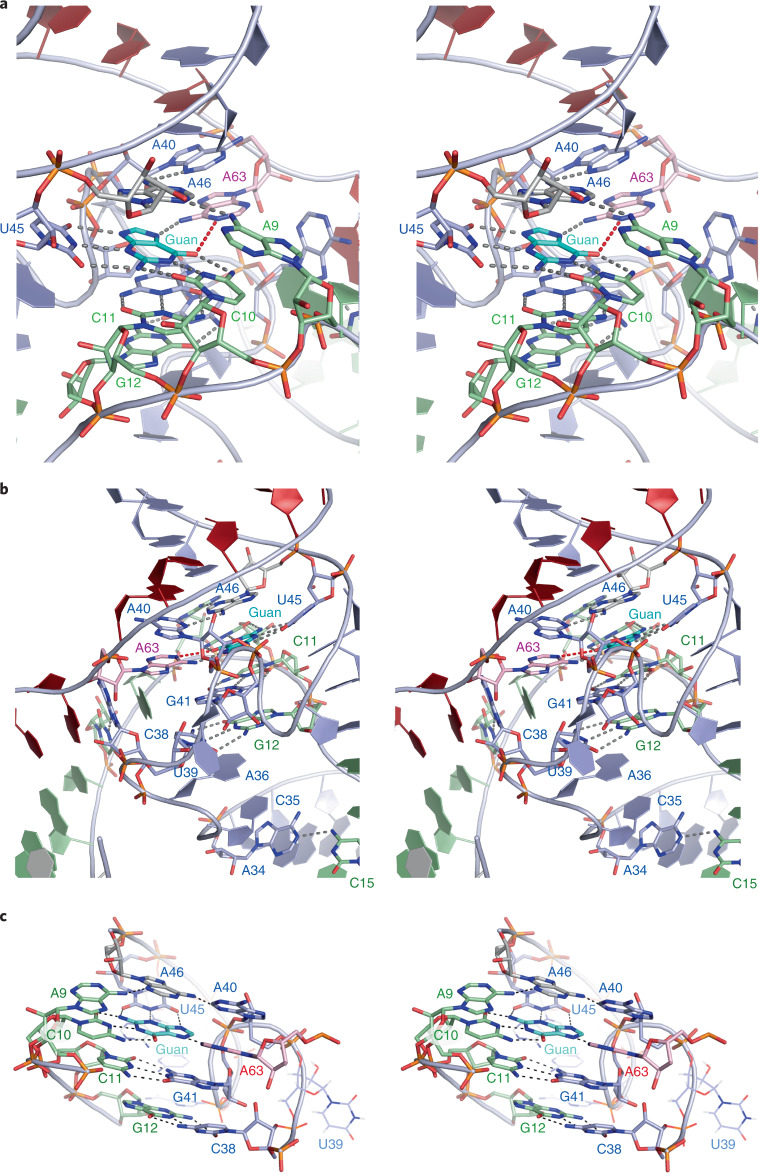
The structure of the core of the MTR1 ribozyme. Parallel-eye stereoscopic images are presented. The bound guanine is colored cyan, and the target A63 is colored magenta. **a**, The core viewed from the minor groove side, with the J12 strand (green) at the front. Note the sequential stacking of the nucleobases A9, C10, C11 and G12 of the J12 strand. **b**, The core viewed from the major groove side, with the J23 strand (blue) at the front. Note the corkscrew-like trajectory of the J23 strand. **c**, The four planes of nucleobase interactions in the core of the ribozyme. The four planes are composed of (from bottom to top) G12•C38, C11•G41, exogenous guanine hydrogen bonded to C10, U45 and A63 and the triple interaction A9•A46•A40.

Helical junctions have two chemically distinct sides with major and minor groove characteristics. Views of each side are presented in Fig. 2a,b. The J12 strand is located on the minor groove side of the junction, while J23 lies on the major groove side. Strand J12 remains approximately helical through the junction, with nucleobases A9 to G12 stacked and a sharp bend between G8 and A9. By contrast, the J23 strand follows a left-handed corkscrew-like path.

**Fig. 3:**
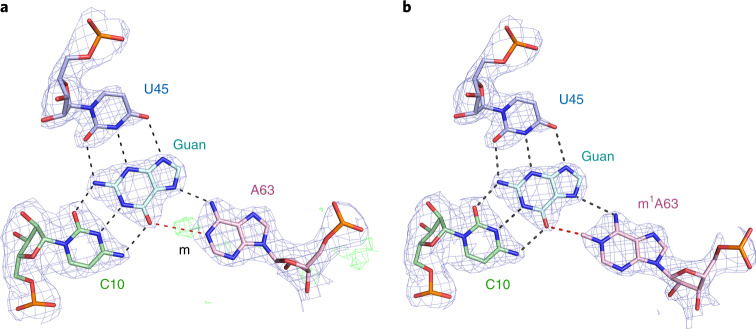
The guanine-binding site and active center of the MTR1 ribozyme. The guanine reaction product forms three hydrogen bonds each with the nucleobases of C10 and U45 and accepts a hydrogen bond at N6 from A63. Hydrogen bonds are colored black. The red broken line denotes the vector linking guanine O6 and A63 N1, that is the expected trajectory of the SN2 reaction. The 2*F*_o_*–F*_c_ map is contoured at 1.5*σ* and colored blue. A63 in the active center has been modeled in two ways. **a**,**b**, The data have been fitted with adenine at position 63, whereupon the methyl group is clearly revealed bound to A63 N1 in the *F*_o_–*F*_c_ omit map (green electron density) (**a**). The peak corresponding to the methyl group is labeled m. Position 63 has been modeled as *N*^1^-methyladenine (m^1^A63) (**b**). This now provides an excellent fit that includes the methyl group bonded to A63 N1.

The central core of the structure can be described simply in terms of four planes of nucleobase interactions sequentially stacked with an average spacing of 3.4 Å (Fig. 2c). These involve the J12 nucleotides G12, C11, C10 and A9 making sequential interactions with successive planes. Nucleotides of the J23 strand are involved in each plane but non-sequentially and in some cases out of order. This arises from the quasihelical nature of the J23 strand, shown clearly in the view of the major groove side (Fig. 2b). The exogenous guanine and the target A63 are intimately involved in the third plane interactions. The four planes of interaction rising upward from the P2 helix planes 1 and 2 are the G12•C38 *cis*-Watson–Crick base pair and the C11•G41 *cis*-Watson–Crick base pair, respectively. The third plane forms where exogenous guanine makes hydrogen bonds to nucleobases C10, U45 and A63 (Fig. 3). Finally, the fourth plane comprises A9•A46•A40, a triple interaction in which the central A46 makes hydrogen bonds to A9 and A40 (Extended Data Fig. 3c).

The third plane includes the reactant and target and constitutes the active center of the ribozyme. This is discussed in the following section.

### The binding of exogenous guanine and the active center

The exogenous guanine (the product of *O*^6^-methylguanine demethylation) is coplanar with the nucleobases of C10, U45 and A63 (Fig. 3) in the third plane of the core. The guanine is central to these interactions; all the hydrogen bonds in this plane involve the guanine nucleobase. The interactions are formed by C10 forming a *cis*-Watson–Crick base pair with guanine with three standard hydrogen bonds, U45 forming a *trans*-Watson–Crick sugar edge base pair with guanine with three hydrogen bonds and A63 forming a *cis*-Watson–Crick–Hoogsteen base pair with guanine with a single hydrogen bond from adenine N6 to guanine N7. The exogenous guanine is almost maximally hydrogen bonded. In addition, it is stacked on both faces, with the C11•G41 base pair comprising the second plane below and with the central A46 of the base triple in the top plane above. Thus, the guanine is rigidly constrained in this site.

**Extended Data Fig. 5:**
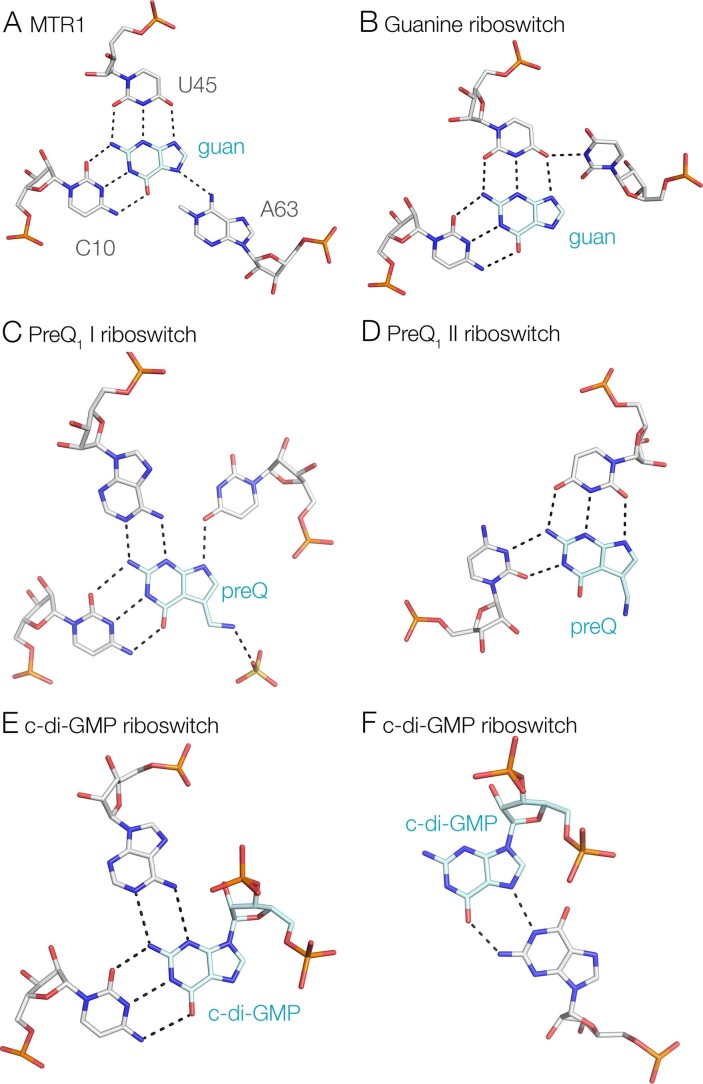
Extended reactions for MTR1 U45C and C10U. Extended reactions for MTR1 U45C and C10U. The same two-piece ribozyme + substrate and conditions used for the unmodified MTR1 ribozyme (Fig. 4) were employed. Radioactively-labeled [5′-^32^P] substrate strand (50 nM) was mixed with 1 µM ribozyme strand in the presence of 50 mM HEPES (pH 7.5), 120 mM KCl, 30 mM MgCl_2_ together with 50 µM *O*^6^-methylguanine at 37 °C and allowed to react for the times indicated. The gel was subject to autoradiography (**a**). Even at the longest time (over 4 days) no adenine methylation was detected for the MTR1 C10U variant. The MTR1 U45C variant achieved a low level of reaction, and fitting the data to a single exponential (**b**) gave a reaction rate *k*_obs_ = 2.7 x10^-5^ min^-1^, with the extent of reaction fixed equal to one, that assumes that the reaction would go to completion as observed for the unmodified MTR1 ribozyme. Source data

The structures of 12 distinct guanine-binding sites have been determined in numerous riboswitches, examples of which are shown in Extended Data Fig. 5; these make an informative comparison with MTR1. In the guanine riboswitch^30^, the binding is closely similar to that observed in MTR1, with cytosine binding the Watson–Crick and uracil binding the sugar edges. Indeed, the formation of what is effectively a Watson–Crick base pair with cytosine is common and is observed in the preQ_1_-I (ref. ^31^), 2′-deoxyguanosine^32^, ppGpp^33^ and cyclic di-GMP^34^ riboswitches. There is more variety in the nature of interactions with the sugar edge, and the Hoogsteen edge is rather less frequently contacted.

The broken red line in Fig. 3a,b shows the expected direction of SN2 attack. This shows that the guanine and A63 are held in the perfect orientation for the methyl transfer reaction. In fact, the crystal structure shows that the reaction has proceeded to completion. If we model an unmodified adenine at A63, we find unassigned electron density 1.4 Å from N1 and colinear with guanine N6, consistent with the presence of a methyl group (Fig. 3a), and no electron density corresponding to methyl attached to O6 of the guanine. Modeling position 63 as *N*^1^-methyladenine gives an excellent fit to the electron density (Fig. 3b). This confirms the conclusion of Höbartner and co-workers^28^ that MTR1 transfers the methyl group from exogenous guanine O6 to A63 N1. It would be expected that *O*^6^-methylguanine would bind into the site in the same way, except that there would be no hydrogen bond possible between C10 N3 and *O*^6^-methylguanine N1. The observation that the reaction has gone to completion incidentally confirms that the ribozyme is fully active.

### The structure of MTR1 is consistent with atomic mutagenesis

Scheitl et al.^28^ performed extensive atomic mutagenesis analysis of the target A63 on methylation activity, and all the results are consistent with the structure we observe in the crystal. Adenine cannot be replaced by either purine or 2-aminopurine. Thus the 6-amino group is important, consistent with the observed hydrogen bond to the exogenous guanine N7. Replacement of either A63 N7 or N3 by CH (that is, deaza substitutions) had little effect on activity, neither of which make interactions observed in the crystal. They also showed that the exocyclic 2-NH_2_ group on the exogenous *O*^6^-methylguanine was required for activity. This makes hydrogen bonds to both C10 and U45 in the crystal structure.

**Fig. 4:**
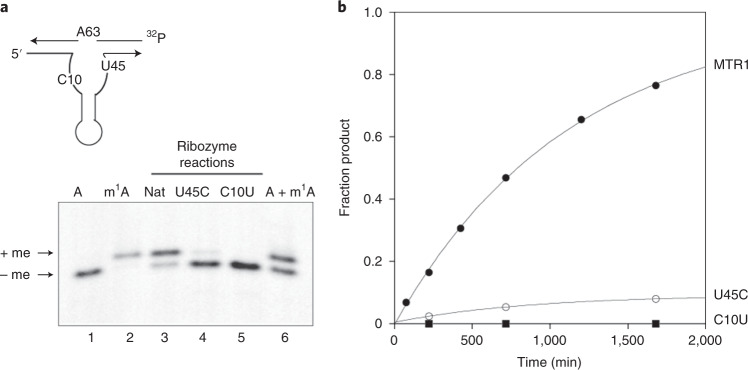
Methyl transfer activity of MTR1 and active center variants. The activity of MTR1 was measured using the electrophoretic assay developed by Scheitl et al.^28^ using the two-piece ribozyme shown schematically. Reactions were performed with 50 nM 5′-[^32^P]-labeled substrate strand incubated with 1 µM ribozyme strand in the presence of 50 mM HEPES (pH 7.5), 120 mM KCl, 5 mM NaCl, 40 mM MgCl_2_ and 50 µM *O*^6^-methylguanine at 37 °C. **a**, Gel electrophoretic separation of substrate and reaction products. The autoradiograph is shown so that only radioactive strands are visible. Tracks 1 and 2 contain chemically synthesized substrate strand with adenine (A) and *N*^1^-methyladenine (m^1^A) at position 63, respectively. Track 6 contains a mixture of the same two oligonucleotides. These serve as markers for the substrate and product of the ribozyme reaction. The products of the ribozyme reaction are shown in tracks 3, 4 and 5. In track 3, the unmodified ribozyme was incubated with radioactive substrate for 28 h, resulting in a substantial conversion to *N*^1^-methyladenine-containing RNA. By contrast, the ribozyme activity was severely impaired by either U45C or C10U variants; Nat, natural; me, methyl. **b**, Plots of reaction progress for unmodified MTR1 and C10U or U45C variants. Aliquots were removed at various times, and the fractions of substrate and product strands were quantified by autoradiography. Reaction progress has been fitted to single exponential functions. Longer time courses have been performed for the C10U or U45C variants (Extended Data Fig. 6) performed in triplicate. The data reveal that the U45C variant has an activity that is 30-fold slower than the unmodified ribozyme. No activity was detectable for the C10U variant even with 4 d of incubation. Source data

**Extended Data Fig. 6:**
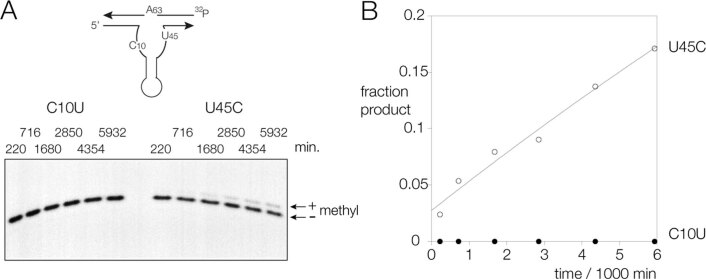
Fluorescent assay used for measurement of alkyl transfer as a function of pH. Fluorescent assay used for measurement of alkyl transfer as a function of pH. **a**. The fluorescent assay using the fluorophore Atto488 (λ_max_ = 500 nm; ε_max_ = 9.0 x 10^4^ M^-1^ cm^-1^) attached to guanine at O6 via an O-[4-(aminomethyl)-benzyl] linkage (Atto488-BG). The reaction transfers the Atto488 to A63 in the substrate strand, generating a fluorescent product band that can be quantified by fluorimaging. A constant quantity of a 19 nt DNA stem-loop 5′-labeled with fluorescein was included in all reactions, and the fluorescence of the product was divided by that of the DNA standard. **b**. An example of a reaction, at pH 6.0. The reaction was performed in 25 mM MES (pH 6.0), 100 mM KCl, 40 mM MgCl_2_ with 1 µM ribozyme strand, 1 µM substrate strand and 50 µM AABG, at 37 °C. The gel was scanned by fluorimaging using excitation by a 473 nm laser with a FITC filter. **c**. The RNA product/DNA ratio is plotted and fitted to a single exponential function, giving a rate of alkyl transfer of *k*_obs_ = 0.077 min^-1^. All such reactions have been performed a minimum of four times. Source data

The nucleobases of C10 and U45 each make three hydrogen bonds to the bound guanine in the crystal. We individually mutated these to C10U and U45C and examined the resulting methyltransferase activity of the ribozyme. For this purpose, we used the assay described by Scheitl et al.^28^ using a two-piece ribozyme–substrate complex with a separate 13-nt substrate strand corresponding to the J31 strand (Fig. 4a). The product strand containing positively charged *N*^1^-methyladenine has a slightly lower electrophoretic mobility than the unmodified substrate. The unmodified MTR1 ribozyme exhibits good methyltransferase activity, that is, a substantial level of product formation with a rate of *k*_obs_ = 9.2 ± 0.3 × 10^−4^ min^−1^ at pH 7.5 (Fig. 4b). By contrast, both C10U and U45C variants exhibited a large impairment of methyltransferase activity, consistent with their role in binding *O*^6^-methylguanine as observed in the crystal structure. Extended reaction time courses showed some formation of *N*^1^-methyladenine with the U45C variant, with a rate of *k*_obs_ = 2.8 ± 0.2 × 10^−5^ min^−1^ that is 30 times slower than the native MTR1 sequence (Extended Data Fig. 6). By contrast, no methyl transfer at all was detected for the C10U variant even after 4 d of incubation (performed in triplicate), indicating a particularly important role for C10.

### A proposed mechanism of methyl transfer

Höbartner and co-workers^28^ showed that the MTR1 ribozyme transfers the methyl group from exogenous guanine O6 to A63 N1, and this is confirmed by our structure. The reaction is therefore expected to involve nucleophilic attack on the methyl carbon atom by A63 N1, leaving guanine with a keto oxygen atom at position 6. In contrast to most SN2 reactions familiar in organic chemistry that are intermolecular, in the context of the ribozyme, the methyl transfer reaction becomes intramolecular. The reactants are held in place, raising their effective local concentration and lowering the entropic activation penalty. Our structure shows that the two reactants are very well aligned for the reaction (Fig. 3). The angle defined by G O6–A63 N1–A63 C6 is 118°, corresponding almost perfectly with the nitrogen *sp*^2^ orbital containing the lone pair of electrons. Thus, local concentration and orientation will confer some catalytic rate enhancement.

**Fig. 5:**
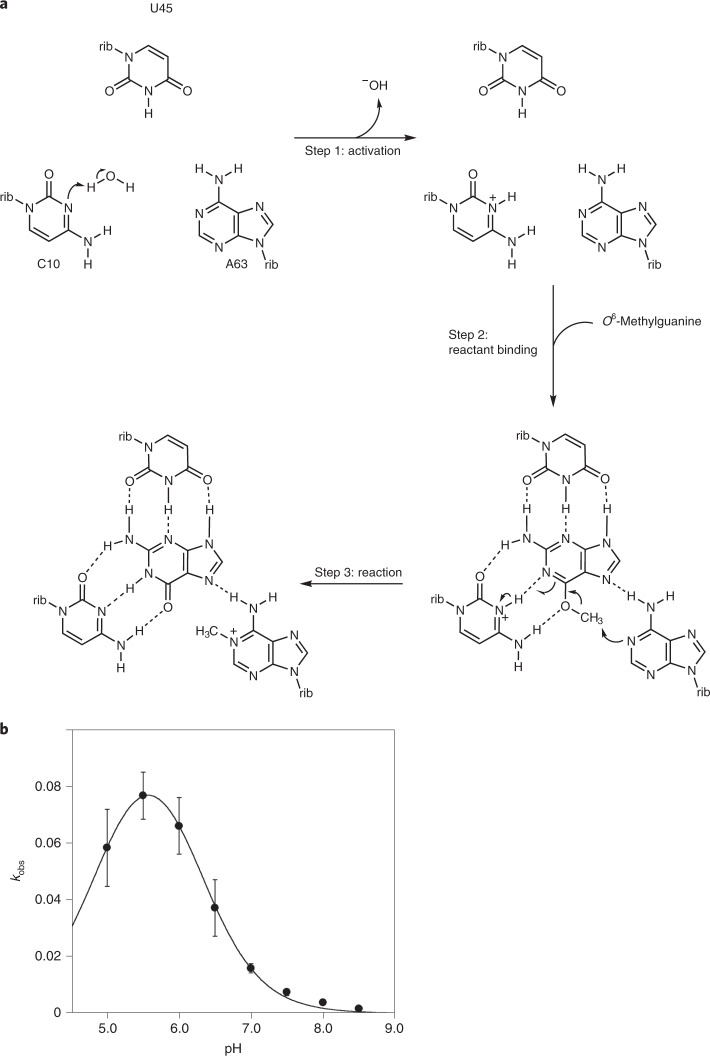
A proposed catalytic mechanism of MTR1. **a**, The chemical mechanism. For clarity, we show the mechanism in three steps. In step 1, the nucleobase of C10 becomes protonated, and in step 2, the *O*^6^-methylguanine becomes bound. However, it is likely that these two steps are coordinated, as the binding will raise the p*K*_a_ of the cytosine. The methyl transfer reaction occurs in step 3 by the nucleophilic attack of A63 N1 on the methyl group of *O*^6^-methylguanine and the coordinated breakage of the guanine O6–C bond. This involves a train of electron transfers, the movement of the proton from C10 N3 to guanine N1 and concomitant shift of the positive charge from C10 to the *N*^1^-methyladenine at position 63. In principle, the guanine can now be released as product, although there is no evidence that this occurs with the present form of the ribozyme. Regeneration of active ribozyme would also require an exchange of the substrate strand to place unmethylated adenine at position 63. The proposed mechanism is fully consistent with the structure of the MTR1 riboswitch and the effect of the substitutions at C10 and U45 on activity (Fig. 4). The complete loss of methylation activity of the C10U variant is fully consistent with the proposed role as general acid in addition to ligand binding. rib, ribose **b**, The pH dependence of MTR1-catalyzed alkyl transfer. The rate of alkyl transfer was measured using a fluorescent assay (Extended Data Fig. 7) multiple times at pH values between 5 and 8.5. The mean values of observed rate constants derived from at least four independent experiments are plotted as a function of reaction pH, where the error bars represent s.d. The data have been fitted to two ionizations (Methods), from which apparent p*K*_a_ values of 5.0 and 6.2 were calculated. The following are the numbers of independent replicates at each pH: pH 5.0, *n* = 5; pH 5.5, *n* = 5; pH 6.0, *n* = 4; pH 6.5, *n* = 5; *n* = 5; pH 7.0, *n* = 5; pH 7.5, *n* = 4; pH 8.0, *n* = 4; pH 8.5, *n* = 4.

Methyl transfer from O6 requires restoration of a single bond between N1 and C6 and thus protonation of N1 in the resulting guanine. This indicates a requirement for a general acid to protonate N1, and the only candidate for this is C10. We therefore propose a possible mechanism for the reaction based on the structure observed in the crystal (Fig. 5a). We suggest that C10 becomes protonated at N3 (step 1) and that this is the form of the ribozyme that binds *O*^6^-methylguanine (in step 2). This permits the formation of three hydrogen bonds between the cytosine nucleobase and *O*^6^-methylguanine, including that from C10 N3 to *O*^6^-methylguanine N1. Methyl transfer (step 3) involves a coordinated movement of the methyl group to A63 N1 and the proton flipping from C10 N3 to guanine N1 within the hydrogen bond, restoring neutral unprotonated C10 and leaving *N*^1^-methyladenine with a positive charge at position 63. In principle, the ribozyme could then be restored by release of guanine, although this would require an exchange of the J31 strand, as the *N*^1^-methyladenine cannot react further. In addition, there is no evidence that product release occurs from the reacted ribozyme, and this was not required by the selection strategy. To summarize, we propose that the reaction is accelerated by the ribozyme using two catalytic strategies, propinquity/orientation and general acid catalysis. Although we have drawn the reaction in three separate steps for clarity, it is likely that these steps could occur in a coordinated manner, and in particular the protonation of C10 and the binding of *O*^6^-methylguanine might be essentially simultaneous. The formation of the base pair would be expected to raise the p*K*_a_ of the cytosine from its normal value of 4.2, as discussed for structured RNA by Moody et al.^35^. The proposed role of the nucleobase of C10 in both substrate binding and general acid catalysis is consistent with our activity data. MTR1 U45C exhibits a low level of activity, but activity in the C10U variant is completely undetectable. This suggests that C10 is doing more than just binding *O*^6^-methylguanine, consistent with an additional role in general acid catalysis.

### The pH dependence of reaction rate

**Extended Data Fig. 7:**
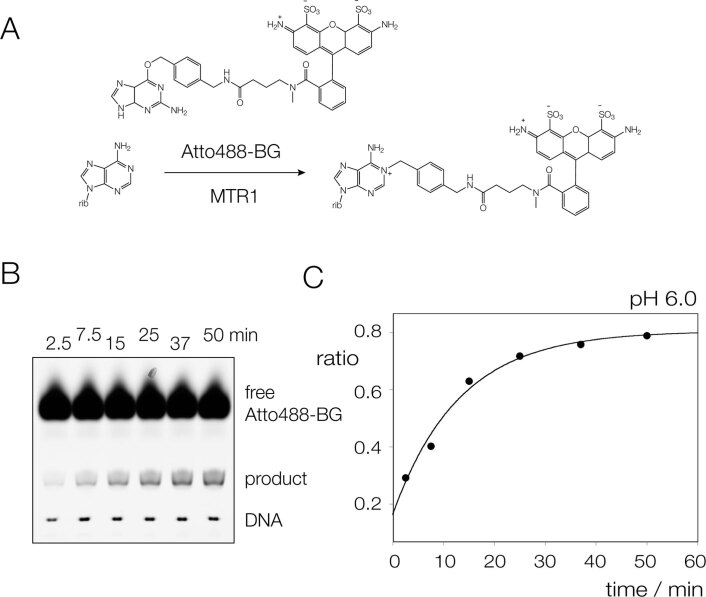
Comparison of guanine binding in the MTR1 ribozyme compared with guanine-related ligand binding in some riboswitch structures. Comparison of guanine binding in the MTR1 ribozyme compared with guanine-related ligand binding in some riboswitch structures. The orientation of the ligand (highlighted in cyan) is similar in all the images. **a**. MTR1 ribozyme (this work). **b**. The guanine riboswitch. PDB ID 6UBU^2^. The binding of the guanine ligand by cytosine and uracil nucleobases is closely similar to that found in MTR1. **c**. The PreQ_1_-I riboswitch. PDB ID 3Q50^3^. The Watson-Crick edge of the pre-queuosine_1_ (preQ_1_) ligand is bound by cytosine, while the sugar edge is bound by the Watson-Crick edge of an adenine at N1 and N6, and makes a single hydrogen bond with a uracil O4. **d**. The PreQ_1_-II riboswitch. PDB ID 4JF2^4^. The sugar edge of the preQ_1_ ligand is bound by a uracil in a similar manner to MTR1, except that the roles of U O2 and O4 are reversed. The Watson-Crick edge is bound by O2 and N3 of a cytosine. **e** and **f**. The cyclic-di-GMP riboswitch. PDB ID 3IRW^5^. The ligand comprises two guanine nucleobases, bound in different environments. The Watson-Crick edge of the guanine shown in part **e** is once again bound to cytosine, while the sugar edge is bound to N1 and N6 of an adenine. By contrast the other guanine (part **f**) is only bound on its Hoogsteen edge, making two hydrogen bonds to the Watson-Crick edge of a guanine at N1 and N2.

Inspection of the structure indicates that binding *O*^6^-methylguanine to the Watson–Crick face of C10 would be stronger if the cytosine nucleobase were protonated at N3 and that the transfer of that proton to N1 of *O*^6^-methylguanine could be an integral part of the catalytic mechanism. This leads to the prediction that the rate of methyl transfer should increase at lower pH, reflecting the p*K*_a_ of C10. We therefore sought to test this prediction by measuring the observed rate of reaction as a function of pH. We devised a new fluorescent assay using the fluorophore Atto488 attached to guanine at O6 via an *O*-(4-(aminomethyl)-benzyl) linkage (Extended Data Fig. 7). This is very similar to the compound used in the original selection of the ribozyme^28^ and would be expected to undergo alkyl transfer, transferring the fluorophore to A63 N1 in the substrate strand. This can be analyzed using electrophoresis on small polyacrylamide gels that are quick to run and are well suited to performing replicate reaction progress time courses at eight pH values between 5.0 and 8.5.

The dependence of reaction rate on pH is plotted in Fig. 5b. The reaction rate is indeed strongly pH dependent, with a maximum rate at pH ~5.6. The rate reduces in a log-linear reaction above pH 6.0, giving an apparent p*K*_a_ value of 6.2. While other explanations are possible, this is consistent with a cytosine with a raised p*K*_a_ due to its local environment and particularly its interaction with the exogenous modified guanine. Below pH 5.6, the reaction rate reduces, and the data can be fitted to two ionizations, with apparent p*K*_a_ values of 5.0 and 6.2. The ionization leading to reduced activity at low pH is plausibly the protonation of A63 N1 that would block the acceptor for alkyl transfer. While kinetic ambiguity prevents the assignment of a particular function to each ionization constant, in this case, we have assumed that the higher ionization constant results from C10 acting as a general acid because its p*K*_a_ shift is readily explicable. These data support (but do not prove) the proposed catalytic mechanism involving proton transfer from N3 of C10, as suggested by the observed structure.

## Discussion

It is interesting to examine the structure of an RNA that has been selected on the basis of catalytic activity. The overall architecture is that of a three-way helical junction, which was to be expected because the P1 and P3 helices were essentially predetermined by the nature of the seed sequence used in selection. The P2 helix formed during the selection process, with Watson–Crick base pairing at the junction distal end. The junction proximal end remains quasihelical, continuing into the core of the junction where the active center is located. Natural three-way helical junctions tend to adopt a structure in which two arms are coaxial and the third is directed away from the axis^36–38^, and the MTR1 junction has followed this general behavior. The structure has relatively few long-range contacts, and the extended P2 helix contains consecutive unpaired nucleotides, forming a bubble. These features are not typical of natural RNA species that are the product of extended evolution in the wild, indicative of a somewhat naive structure and perhaps showing the limits of in vitro selection. The critical region of the RNA lies in the core of the junction, where four planes of nucleobase interaction lie between the J12 and J23 strands, creating the environment that binds the exogenous *O*^6^-methylguanine in such a way that it acts site specifically as a methyl donor. The coaxial stacking of the P2 and P3 helices and the divergence of the P1 helix makes a pronounced kink in the J31 strand at the position of A63, ‘presenting’ this nucleobase for attack. The local structure immediately suggests a catalytic mechanism for the ribozyme that we discuss below.

Guanine can donate up to four protons and has three potential hydrogen bond acceptors. In MTR1, guanine forms seven hydrogen bonds to the RNA and is bound predominantly on its Watson–Crick edge by C10 and its sugar edge by U45. In the MTR1 ribozyme, the nucleobases of C10 and U45 hold the exogenous guanine tightly, directing the O6 on the Hoogsteen edge toward the target A63. This is an important part of the ribozyme action, as we discuss further in the following section.

Extrapolating the structure of the complex of the ribozyme with exogenous guanine to the expected prereaction complex shows that a methyl group attached to O6 of guanine would be almost perfectly aligned for nucleophilic attack by N1 of A63. Part of the catalytic rate enhancement therefore arises from effective local concentration and orientation. A second element in the catalysis likely comes from general acid catalysis by the cytosine nucleobase of C10 that transfers a proton attached to its N3 to N1 of the exogenous *O*^6^-methylguanine. This is strongly suggested by the structure of the complex in the crystal and supported by the pH dependence of the reaction rate. To our knowledge, there is no other example to date of a selected ribozyme using general acid catalysis, for example, in the three other structures known for selected ribozymes^13,14,27^.

The intramolecular nature of the reaction raises a question about how the nucleophilic attack is initiated. This requires the highest occupied molecular orbital of the nitrogen nucleophile (that is, the lone pair-containing *sp*^2^ orbital) to overlap the lowest unoccupied molecular orbital of the carbon that is the σ* orbital of the C–O bond. In the structure of the product observed in the crystal, the distance between the A63 N1 and G O6 atoms is 4.1 Å, so that the distance from *O*^6^-methylguanine methyl carbon to A63 N1 will be ~ 3 Å. Initiation of the reaction requires a closer approach for effective *sp*^2^–σ* orbital overlap. The exogenous guanine (and therefore the *O*^6^-methylguanine) appears be held in place so rigidly that it seems more probable that the nucleobase of A63 would be required to move closer while remaining in plane. A63 lies at the junction between P1 and P3, and A63 is consequently unstacked on its lower face. Its O2′ donates a hydrogen bond to A37 N3, but deletion of this hydroxyl group led to no loss of activity^28^. On the upper side, A63 is not directly stacked onto G62 but rather on A40, which is part of the triple base interaction. It therefore seems that the environment of A63 may be more flexible than that of the *O*^6^-methylguanine. As a working hypothesis, we propose that the methyl transfer reaction might be coupled to a local conformational change that allows the N1 of A63 to move closer, perhaps by a combination of rotation and translation of the nucleobase and potentially involving a reorientation of the P1 helix at the junction.

The MTR1 ribozyme is relatively slow even at the low pH maximum, but given that this was the product of only 11 rounds of selection^28^, perhaps high catalytic efficiency should not be expected. By contrast, natural ribozymes were likely refined over millions of years of evolution that could have improved their catalytic activity. Nevertheless, MTR1 demonstrates that catalysis of methyl and alkyl transfer by RNA is possible, and this work indicates that a relatively sophisticated catalytic mechanism can evolve. This lends new credence to the concept of the RNA world in the development of early life on the planet.

## Methods

### Chemicals

*O*^6^-Methylguanine (O837914) was obtained from Macklin. Atto488 NHS-ester was obtained from Atto-Tec, and *O*-(4-(aminomethyl)-benzyl)guanine was obtained from Biosynth Carbosynth.

### RNA transcription

A 69-nt MTR1 RNA for crystallization (all sequences are written 5′ to 3′) GCGGGCUGACCGACCCCCCGAGUUCGCUCGGGGACAACUAGACAUACAGUAUGAAAAUACUGAGCCCGC

that was based on the CA13 selected ribozyme^28^ was transcribed in vitro using T7 RNA polymerase at 37 °C. The RNA was purified by electrophoresis in a polyacrylamide gel in the presence of 7 M urea. The full-length RNA was visualized by UV shadowing, excised and electroeluted (Elutrap Electroelution System, GE Healthcare) in 0.5× TBE buffer for 12 h at 200 V at 4 °C. The RNA was precipitated with isopropanol, washed with 70% ethanol and dissolved in water.

### Chemical synthesis of RNA

Oligonucleotides were synthesized using *tert*-butyldimethyl-silyl (*t*-BDMS) phosphoramidite chemistry^39^, as described in Wilson et al.^40^. This was implemented on an Applied Biosystems 394DNA/RNA synthesizer using UltraMILD ribonucleotide phosphoramidites with 2′O-*t*-BDMS O2′-protection^41,42^ (Link Technologies).

Ribo-oligoribonucleotides, including *N*^1^-methyladenine (Glen Research), were deprotected using anhydrous 2 M ammonia in methanol (Sigma-Aldrich) for 24 h. Unmodified RNA was deprotected in 1:1 ammonia:methylamine at 60 °C for 20 min. Dried oligoribonucleotides were redissolved in 115 μl of anhydrous DMSO, 60 µl of triethylamine and 75 μl of triethylamine trihydrofluoride to take off *t*-BDMS groups and shaken in the dark at 65 °C for 2.5 h before butanol precipitation.

The following RNA sequences were used in the kinetic assay of methyltransferase activity: MTR1, GCGGGCUGACCGACCCCCCGAGUUCGCUCGGGGACAACUAGACAUACAGUAU; MTR1 C10U, GCGGGCUGAUCGACCCCCCGAGUUCGCUCGGGGACAACUAGACAUACAGUAU; MTR1 U45C, GCGGGCUGACCGACCCCCCGAGUUCGCUCGGGGACAACUAGACACACAGUAU; MTR substrate, AUACUGAGCCCGC; MTR product, AUACUG(m^1^A)GCCCGC (m^1^A denotes the position of *N*^1^-methyladenine).

The following RNA sequences were used in the study of alkyltransferase activity using fluorescence: MTR1, GCGUCUUAAGGCUGACCGACCCCCCGAGUUCGCUCGGGGACAACUAGACAUACAGUAUGUCACG; MTR substrate, CGUGACAUACUGAGCCUUAAGACGC.

### Crystallization, structure determination and refinement

A number of constructs were put into crystallization trials, using either one or two strands, and no contructs based on two strands yielded diffracting crystals. Using constructs based on a single strand, both P1 and P3 could be capped with a GAAA loop, and different lengths for stems P1 and P3 from 5 to 8 base pairs were explored. The sequence shown above with P1 and P3 helices of 6 base pairs and a GAAA loop on P3 yielded well-diffracting crystals that were used in this study.

A solution of 0.5 mM MTR1 RNA (69 nt) in 5 mM HEPES (pH 7.5) and 100 mM KCl was slowly cooled from 95 °C to 20 °C followed by addition of MgCl_2_ to a final concentration of 5 mM. *O*^6^-Methylguanine was added at a final concentration of 5 mM. Drops were prepared by mixing 0.8 μl of the RNA–ligand complex with 0.8 μl of a reservoir solution composed of 40 mM sodium cacodylate (pH 7.0), 0.08 M KCl, 0.02 M BaCl_2_, 30% (vol/vol) (+/–)-2-methyl-2,4-pentanediol and 0.012 M spermine tetrahydrochloride, and crystals were grown at 18 °C by hanging drop vapor diffusion. Crystals appeared after 3 d and were frozen by mounting in nylon loops and rapid immersion into liquid nitrogen.

Diffraction data were collected at beamline BL02U1 of the Shanghai Synchrotron Radiation facility at a wavelength of 0.97918 Å at 100 K. Data were processed using Aquarium^43^. The resolution cutoff for the data was determined from the the Pearson correlation coefficient CC_1/2_ and the density map^44^. Crystals grew in space group *P*222_1_ with unit cell dimensions of *a* = 50.38 Å, *b* = 52.5 Å and *c* = 99.9 Å. Eleven barium(II) sites were identified and exploited in single-wavelength anomalous dispersion phasing using the AutoSol function of PHENIX^45^. Density modification using RESOLVE led to an interpretable electron density map (Supplementary Fig. 1).

The structure was determined in PHENIX 1.19 (ref. ^45^). Models were adjusted manually in Coot 0.9.6 (ref. ^46^) and underwent several rounds of adjustment and optimization using Coot^46^, phenix.refine and PDB_REDO^47^. The geometry of the model and its fit to electron density maps were monitored with MOLPROBITY^48^ and the validation tools in Coot. Atomic coordinates and structure factor amplitudes have been deposited at the PDB under accession code 7V9E. Crystallographic statistics are presented in Supplementary Table 1.

### Kinetic assay of MTR1 methyl transfer activity

Single-turnover assays were performed following the method of Sheitl et al.^28^ with minor modifications. Briefly, 20 pmol of MTR1 ribozyme strand was annealed to 1 pmol of 5′-[^32^P]-labeled target RNA in 8 μl of buffer (150 mM KCl, 6.25 mM NaCl and 62.5 mM HEPES (pH 7.5)) by rapid cooling from 80 °C, to which was added 2 µl of 400 mM MgCl_2_. This was equilibrated at 37 °C, and the reaction was initiated by addition of an equal volume of 100 µM *O*^6^-methylguanine in 120 mM KCl, 5 mM NaCl and 50 mM HEPES, pH 7.5. Reactions were incubated under mineral oil to prevent evaporation, and 2-µl aliquots were taken at time intervals and added to 13 µl of 95% formamide and 50 mM EDTA stop mix. Samples (5 µl) were electrophoresed ~35 cm in a 20% denaturing polyacrylamide gel, band intensities were quantified by phosphorimaging, and reaction progress was fitted to the single exponential function

$$y = y_0 + A\left( {1 - e^{ - kt}} \right)$$

where *A* is the amplitude of the reaction, *k* is the rate of reaction, and *t* is the elapsed time. The rates and uncertainties given in the text are the mean and s.d. of three or more independent experiments.

### Kinetic assay of MTR1 activity using fluorescence

We sought to develop a more convenient assay based on fluorescence instead of a shift in mobility of the modified RNA and selected the fluorophore Atto488 (*λ*_max_ = 500 nm; *ε*_max_ = 9.0 × 10^4^ M^−1^ cm^−1^) attached to guanine at O6 via an *O*-(4-(aminomethyl)-benzyl) linkage (Atto488-BG). The reaction transfers the Atto488 to A63 in the substrate strand, generating a fluorescent product band that can be quantified by fluorimaging using excitation by a 473-nm laser and a fluorescein isothiocyanate (FITC) filter to collect emission (Extended Data Fig. 7). A constant quantity of a 19-nt DNA stem-loop labeled with FITC on the 5′-end (Flu-ATATATATGAAATATATAT) was included in all reactions, and the fluorescence of the product was divided by that of the DNA standard.

To synthesize Atto488-BG, 1.5 µmole of *O*-(4-(aminomethyl)-benzyl)guanine and 0.25 µmole of Atto488 NHS-ester were dissolved in 20 µl of DMSO, and 0.5 µl of triethylamine was added. Following overnight incubation at room temperature, 480 µl of 0.1 M TEAA in water was added, and the reactants were separated by HPLC on a C18 reversed-phase column using an acetonitrile gradient. The reaction was quantitative as judged by the absorbance of the fluorophore. Fractions containing Atto488-BG were dried, dissolved to 200 µM in water and stored at −20 °C.

### Determination of reaction rate as a function of pH

Single-turnover assays were performed using longer RNA strands such that helices P1 and P3 each had 12 base pairs. Reactions were performed as described above, except that 20 pmol of ribozyme strand was annealed to 20 pmol of target RNA in 8 μl of 125 mM KCl and 10 µM Flu-DNA by rapid cooling from 80 °C, to which 2 µl of 400 mM MgCl_2_ was added; the reaction was initiated by addition of an equal volume of 100 µM Atto488-BG in 100 mM KCl and 50 mM buffer. The buffers used were MES (pH 5.0 to 6.5), MOPS (pH 7.0 and pH 7.5) and TAPS (pH 8.0 and pH 8.5). The rates and uncertainties for each pH are the mean and s.d. of at least four independent experiments.

The observed pH dependence of the reaction was fitted to a two-ionization model

$$y = k_{{\textrm{int}}}/(1 + 10^{B - {{{\mathrm{pH}}}}} + 10^{{{{\mathrm{pH}}}} - A} + 10^{B - A}),$$

where *k*_int_ is the intrinsic rate of the reaction, *A* is the p*K*_a_ of a moiety where protonation is required for activity (such as a general acid), and *B* is the p*K*_a_ of a moiety where the deprotonated form is active. While kinetic ambiguity prevents the assignment of a particular function to each ionization constant, in this case, we have assumed the higher ionization constant results from C10 acting as a general acid because its p*K*_a_ shift is readily explicable.

### Reporting Summary

Further information on research design is available in the Nature Research Reporting Summary linked to this article.

## Online content

Any methods, additional references, Nature Research reporting summaries, source data, extended data, supplementary information, acknowledgements, peer review information; details of author contributions and competing interests; and statements of data and code availability are available at 10.1038/s41589-022-00982-z.

## Supplementary information

Supplementary Information Supplementary Fig. 1 and Table 1.

Reporting Summary

## Data Availability

The coordinates of MTR1 have been deposited in the PDB under the identifier 7V9E. Source data are provided with this paper.

## Source data

Source Data Fig. 4 Unprocessed gel.
Source Data Extended Data Fig. 5 Unprocessed gel.
Source Data Extended Data Fig. 6 Unprocessed gel.
