# Author Correction: Structure and mechanism of a methyltransferase ribozyme

**DOI:** 10.1038/s41589-022-01073-9

**Published:** 2022-06-06

**Authors:** Jie Deng, Timothy J. Wilson, Jia Wang, Xuemei Peng, Mengxiao Li, Xiaowei Lin, Wenjian Liao, David M. J. Lilley, Lin Huang

**Affiliations:** 1 Guangdong Provincial Key Laboratory of Malignant Tumor Epigenetics and Gene Regulation, Guangdong-Hong Kong Joint Laboratory for RNA Medicine, Sun Yat-Sen Memorial Hospital, Sun Yat-Sen University, Guangzhou, China; 2 Medical Research Center, Sun Yat-Sen Memorial Hospital, Sun Yat-Sen University, Guangzhou, China; 3 Cancer Research UK Nucleic Acid Structure Research Group, MSI/WTB Complex, The University of Dundee, Dundee, UK; 4 College of Life Sciences, Guangzhou University, Guangzhou, China; 5 Department of Urology, Sun Yat-Sen Memorial Hospital, Sun Yat-Sen University, Guangzhou, China

**Keywords:** Biocatalysis, RNA

Correction to: *Nature Chemical Biology* 10.1038/s41589-022-00982-z, published online 17 March 2022.

In the version of this article initially published, there was an error in the first paragraph of Results and the Data availability section, where the PDB ID, now reading PDB ID “7V9E” originally read “7V9B.” The changes have been made to the HTML and PDF versions of the article.

