# Supplementary material for: Structure and mechanism of a methyltransferase ribozyme
Source: Nat Chem Biol. 2022 Mar 17;18(5):556–64. doi: 10.1038/s41589-022-00982-z (PMC9050513; doi:10.1038/s41589-022-00982-z)
Supplement: Supplementary file 1 — Supplementary Fig. 1 and Table 1. [file 41589_2022_982_MOESM1_ESM.pdf]

nature portfolio

https://doi.org/10.1038/s41589-022-00982-z

Supplementary information

Structure and mechanism of a methyltransferase ribozyme

In the format provided by the authors and unedited

Structure and mechanism of a methyl transferase ribozyme

J. Deng et al

Supplementary Information

SUPPLEMENTARY FIGURES

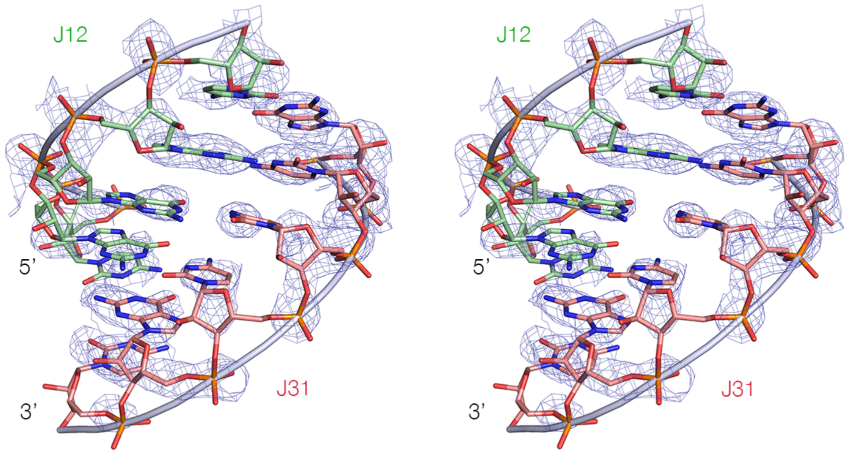

Supplementary Figure 1. Parallel-eye stereoscopic view of the MTR1 P1 helix with the experimental electron density map contoured at 1.5 σ.

SUPPLEMENTARY TABLE

Supplementary Table 1

	MTR1 ribozyme (barium chloride)
**Data collection**	
Space group	P 222_1
Cell dimensions	
a, *b*, *c* (Å)	50.38, 52.51, 99.94
α, β, γ (°)	90, 90, 90
Resolution (Å)	50.38 - 2.30 (2.43 - 2.30)*
R_{merge}	0.091 (0.622)
$I / \sigma I$	13.0 (2.5)
CC(1/2)	99.7 (75.0)
Completeness (%)	98.5 (99.8)
Redundancy	8.1 (5.5)
Wavelength	0.97918
**Refinement**	
Resolution (Å)	24.43 - 2.30 (2.41 - 2.30)
No. reflections	12036 (1199)
R_{work} / R_{free}	0.244/0.271 (0.330/0.354)
No. atoms	
RNA	1440
Ligand/ion	27
Water	32
B-factors	
RNA	65.04
Ligand/ion	50.08
Water	53.69
R.m.s. deviations	
Bond lengths (Å)	0.004
Bond angles (°)	0.715

One crystal was used.

*Values in parentheses are for highest-resolution shell.

CC(1/2) is the percentage of correlation between intensities from random half-data sets as defined in Karplus and Diederichs [6].

Table 1. Data collection and refinement statistics (SAD). The coordinates have been deposited in the PDB with ID 7V9B.
