# Supplementary figures and images for: Structure and mechanism of a methyltransferase ribozyme
Source: Nat Chem Biol. 2022 Mar 17;18(5):556–64. doi: 10.1038/s41589-022-00982-z (PMC9050513; doi:10.1038/s41589-022-00982-z)

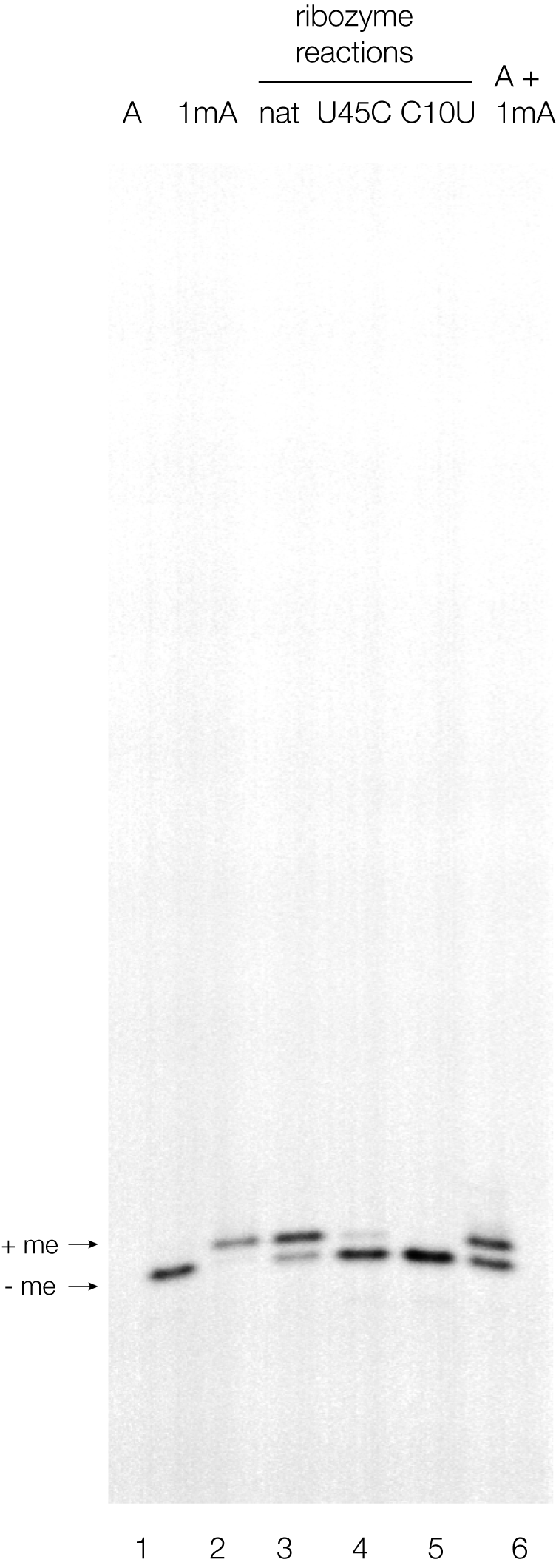

Supplement: Source Data Fig. 4 — Unprocessed gel. [file 41589_2022_982_MOESM3_ESM.tif]

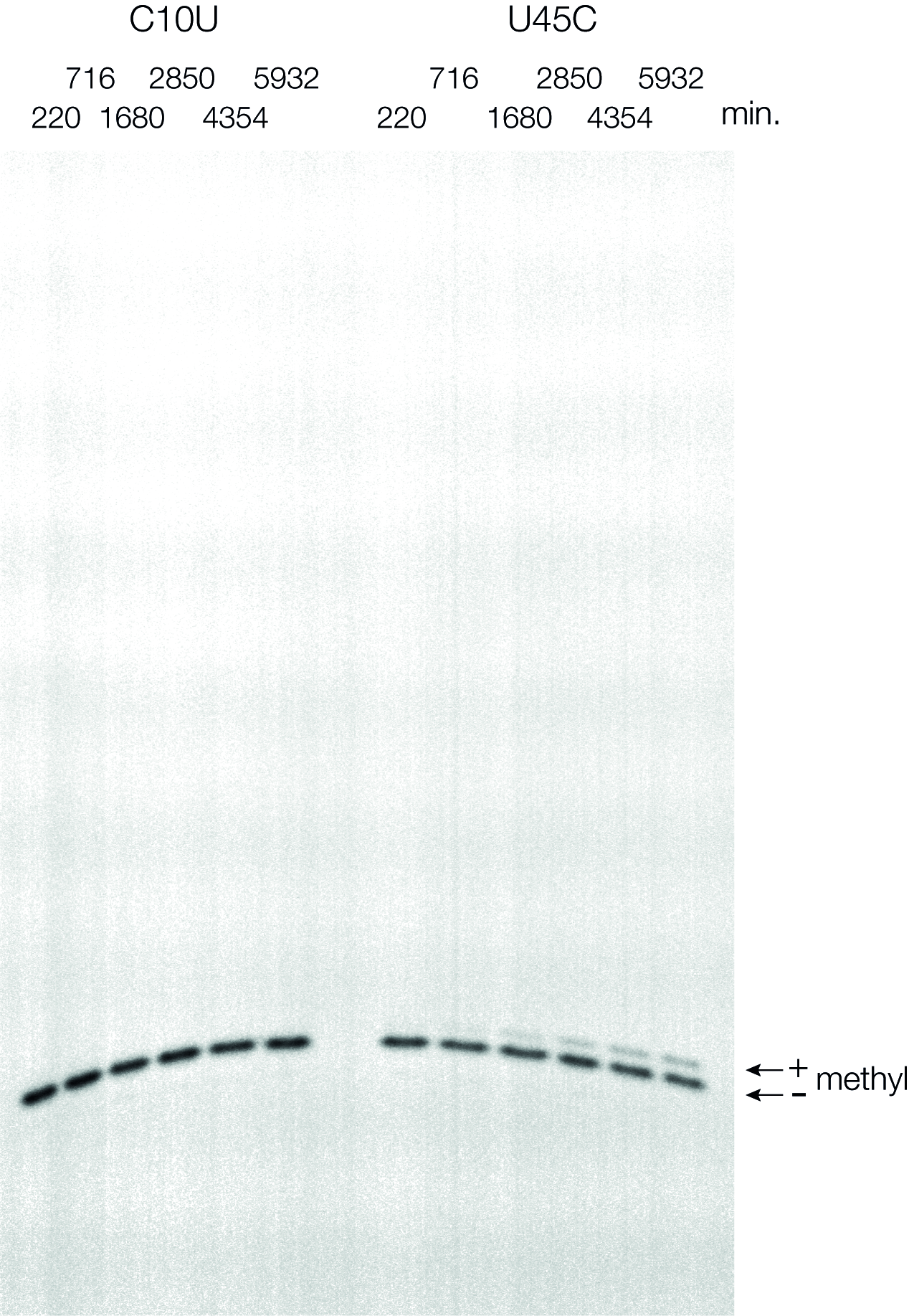

Supplement: Source Data Extended Data Fig. 5 — Unprocessed gel. [file 41589_2022_982_MOESM4_ESM.tif]

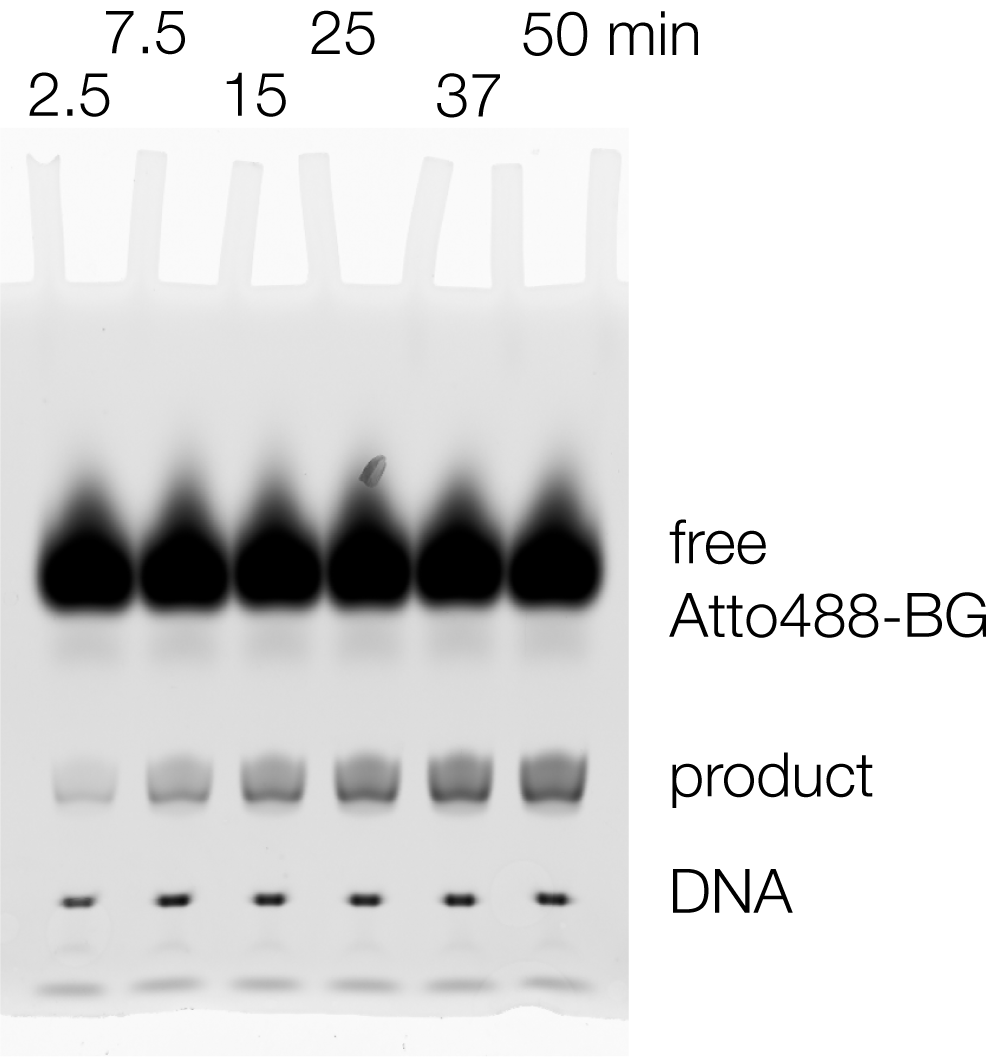

Supplement: Source Data Extended Data Fig. 6 — Unprocessed gel. [file 41589_2022_982_MOESM5_ESM.tif]
